# Adherence to the eat-lancet planetary health diet and sustainability impacts of the Swiss diet

**DOI:** 10.1007/s00394-026-04001-y

**Published:** 2026-05-15

**Authors:** Flurina Suter, Anita Frehner, Adrian Muller, Diego Moretti, Sabine Rohrmann, Giulia Pestoni

**Affiliations:** 1 Division of Chronic Disease Epidemiology, Epidemiology, Biostatistics and Prevention Institute (EBPI), University of Zurich, Zurich, Switzerland; 2 Department of Food System Sciences, Research Institute of Organic Agriculture FiBL, Frick, Switzerland; 3 Nutrition Group, Swiss Distance University of Applied Sciences (FFHS), University of Applied Sciences and Arts of Southern Switzerland (SUPSI), Zurich, Switzerland

**Keywords:** EAT-Lancet, Planetary health diet, Diet quality, Sustainability, Switzerland, menuCH

## Abstract

**Purpose:**

Diets low in quality represent a large risk for global mortality, morbidity, and environmental deterioration. In 2019, the EAT-Lancet commission published a universal reference diet, the ‘planetary health diet’ (PHD), focusing on human health and sustainability. We aimed to compare the average Swiss diet, four Swiss dietary patterns, and the sex- and language-specific dietary habits with the PHD with respect to adherence and sustainability.

**Methods:**

Data of the cross-sectional population-based National Nutrition Survey menuCH (2014–2015, *n* = 2057) were used. Food consumption was assessed with 24-hours dietary recalls. Using the multiple source method, the usual dietary intake was determined. Sustainability impacts were examined by determining Greenhouse Gas emissions, total land and grassland use, the Social Hotspots Index, and diet’s costs. Adherence and sustainability of the Swiss diet were analyzed descriptively.

**Results:**

Participants consumed on average more red meat, dairy products, and added sugar and less whole grains, vegetables, legumes, nuts, and unsaturated fats than recommended by the PHD. None or few of the participants adhered to the whole grains (0.0%), legumes (0.0%), and nuts (5.1%) recommendations, whereas nearly all adhered to the tubers and starchy vegetables (97.7%) and saturated fats (99.9%) recommendations. Overall, diets low in animal-source products tended to have reduced Greenhouse Gas emissions and land use, whereas diets high in fruits and vegetables tended to have higher costs and risks of adverse social impacts. In the subgroup analyses, closer adherence to the PHD was seen among females and among participants following the Prudent dietary pattern, while the differences in sustainability impacts were generally small among subgroups.

**Conclusion:**

A dietary shift decreasing consumption of animal-based protein sources and increasing consumption of plant-based foods would be beneficial for Swiss residents’ health and lower the environmental footprint of the average Swiss diet. Policy measures should incentivize this dietary shift towards a healthier and more sustainable diet, especially among males and individuals following a traditional or Western dietary pattern, as they have the highest potential for improvement.

**Supplementary Information:**

The online version contains supplementary material available at https://doi.org/10.1007/s00394-026-04001-y.

## Introduction

Human health is influenced by multiple factors and behaviors, such as genetics, social status, environment, and diet [1]. Diets low in quality represent a larger risk for global mortality and morbidity than tobacco smoke, alcohol and drug use, and unsafe sex combined [2]. In 2023, globally, 12.2% of all deaths could be attributed to poor dietary habits, being the fifth most common risk factor for early death [3]. In addition, the current food system is a main driver of environment deterioration, as it has been estimated to account for about 30% of all Greenhouse Gas (GHG) emissions and 80% of fresh water use [4, 5]. Consequently, a transformation of our global food system is urgently needed, not only to improve human health but to also enhance environmental sustainability [6].

In January 2019, the EAT-Lancet commission published a universal, healthy reference diet, the ‘planetary health diet’ (PHD) [6]. The PHD provides recommended daily amounts for 14 food components and promotes the consumption of a variety of plant-based foods, low intake of animal source foods, and limited intake of refined grains, ultra-processed foods, and sugars [6]. The PHD focuses not only on human health but also on the environment by proposing a diet rich in foods that can be obtained with a more sustainable food production.

In previous studies, countries have compared their national average diet with the PHD, either considering the total average food consumption in the country or creating a score reflecting adherence to the PHD. In general, in Western countries a rather low to moderate adherence to the PHD was observed, often with an overconsumption of animal-based proteins (such as meat, poultry, and eggs), dairy products, added sugars, and discretionary food and an underconsumption of nuts, vegetables, fruits, and legumes [7–13].

To the best of our knowledge, there is no study investigating adherence to the PHD in Switzerland. Therefore, the aim of this study was to investigate the adherence of the Swiss diet to the PHD and estimate its sustainability impacts. By making use of the first National Nutrition Survey, menuCH, we aimed to compare the average Swiss diet, the four main Swiss dietary patterns published by Krieger et al. [14], and the sex- and language-specific diets to the PHD. In addition, we aimed to explore the sustainability impacts of the average Swiss diet overall and by the different subgroups.

## Methods

Our study is a secondary analysis of the menuCH survey data (2014–2015, *n* = 2057, Switzerland), which is the first and most recent national nutrition survey conducted in Switzerland, and the findings of the analysis by Krieger et al. [14]. The structure of this article followed the STROBE-nut guidelines [15].

### Study design and participants of menuCH

From January 2014 until February 2015 the cross-sectional population-based study, menuCH, was run in ten centers across Switzerland [14, 16, 17]. Participants filled out one questionnaire [18] and answered two 24-h dietary recalls (24HDR) [16].

A representative sample of the Swiss population was drawn with the assistance of the Federal Statistical Office (FSO) [17]. The target sample of 4,627,878 Swiss residents represented Swiss adults of both sexes (female, male), five age categories (18–29, 30–39, 40–49, 50–64, and ≥ 65 years old), the twelve most populated Swiss cantons of the seven major regions, and the three main language regions in Switzerland (CH-German, CH-French, and CH-Italian) [16, 19].

The source population included a stratified, random sample of 13,606 residents, of which 5496 were eligible and successfully contacted [17]. Additional 3439 participants were excluded, since they were non-responders or had an incomplete dietary assessment, respectively. Hence, 2057 individuals were eventually included in the present analyses. A participant flowchart of the menuCH survey is provided in the Supplementary Material (Fig. S1).

All participants gave written informed consent. The study followed the ethical regulations of the Declaration of Helsinki. The ethics committee of Lausanne (protocol 26/13) and the corresponding cantonal ethics committees approved the menuCH survey protocol. The international standard randomized controlled trial number (ISRCTN) of the registered menuCH survey is 16,778,734 [20].

## Data collection in the menuCH survey

The self-administered questionnaire contained questions about the participants’ lifestyle, dietary habits, and sociodemographic factors [18], such as sex, age, and language region. Information on avoidance of a specific food component (eggs, meat, fruits, vegetables, fish, sea food, cheese, milk, other milk products, nuts, or wheat products) was taken from the menuCH questionnaire [18].

Two non-consecutive 24HDR interviews collected data on the participants’ diet. Intake of dietary supplements was not assessed in the 24HDR. The interviews were distributed over all seasons and weekdays and were performed by trained dietitians [17]. The study used the trilingual Swiss version (0.2014.02.27) of the validated and automated software GloboDiet^®^ (formerly EPIC-Soft^®^, International Agency for Research on Cancer (IARC), Lyon, France [21, 22]; adapted by the Federal Food Safety and Veterinary Office, Bern, Switzerland). The first 24HDR was conducted in a study centre and the second one two to six weeks later via telephone [16]. A book including pictures of about 60 household measures and 119 series of five to six graduated portion-sized pictures [23] was given to the participants to ease the quantification of consumed amount of food [24]. Subsequently, the data were cleaned and screened according to IARC’s guidelines using an updated version of GloboDiet^®^ (0.2015.09.28) to ensure high data quality [25]. Finally, documented ingredients, foods, and recipes were linked to the most suitable item listed in the Swiss Food Composition Database [26] by making use of the matching tool FoodCase (Premotec GmbH, Winterthur, Switzerland).

## The four main Swiss dietary patterns

Using the data of the menuCH study, Krieger et al. described four common Swiss dietary patterns [14]: the ‘Swiss traditional’ diet represented the average diet with higher chocolate and dairy intakes; the ‘Western-soft drinks’ and ‘Western-alcohol’ diets were both characterized by a high consumption of red and processed meat. The ‘Western-soft drinks’ diet was additionally characterized by a high consumption of non-alcoholic beverages, whereas the ‘Western-alcohol’ diet by a high consumption of alcoholic beverages, cereals, and starchy food; the ‘Prudent’ diet represented a diet with high intakes of fruits, vegetables, white meat, and fish.

## Planetary health diet by the EAT-Lancet commission

The EAT-Lancet Commission established a universal reference diet, the PHD, that optimizes human and environmental health simultaneously [6]. It included 14 food components (whole grains, tubers and starchy vegetables, vegetables, fruits, dairy foods, red meat, poultry, eggs, fish, legumes, nuts, unsaturated fats, saturated fats, and added sugars). For each of these 14 food components, the PHD lists a recommended daily amount including a lower and upper boundary in grams (except for the food component ‘whole grains’ for which no range is provided) [6].

## Operationalization of the PHD in menuCH

Table 1 and S1 describe the 14 PHD food components with their target value and their lower or upper range limit. Additionally, Tables 1 and S1 show the menuCH food items and food groups included in each of the 14 food components. For all food components of the PHD, the amount of consumed food or drinks in grams per interview day and menuCH participant were calculated.

**Table 1:** Description of the 14 recommended food components of the planetary health diet with their target value (g/day) and lower or upper range limit (g/day) based on a diet of 2500 kcal/day

Dietary component	Target amount (g/day)	Lower/upper amount limit (g/day)^a^	Food items based on menuCH 24 HDR
*Whole grains*			
1 Rice, wheat, corn, and other^b^	464	≥ 464	Cereals, cereal products, and grains with a carbohydrate-to-fiber ratio < = 10:1 or including ‘whole grain’ in the food item’s name
*Tubers or starchy vegetables*			
2 Potatoes and cassava	50	≤ 100	Potatoes and other starchy root tubers
*Vegetables*			
3 All vegetables	300	≥ 200	Vegetables, vegetables juices, and vegetable soups
*Fruits*			
4 All Fruits	200	≥ 100	Fruits and fruit juices
*Dairy foods*			
5 Whole milk or equivalent^c^	250	≤ 500	Milk, milk-based desserts, cottage cheese, curd, yoghurt, sour milk products, cheese, (fermented) milk-based drinks, cream, milk-based ice cream, and butter
*Protein sources*			
6 Beef, lamb, and pork	14	≤ 28	Red meat including mammals, game, offal, unspecified and mixed red meat, meat products
7 Chicken and other poultry	29	≤ 58	Poultry, unspecified and mixed poultry meat, and meat products based on poultry
8 Eggs	13	≤ 25	Eggs and egg products
9 Fish	28	≥ 28	Fish, seafood, amphibians, and reptiles
10 Legumes^b^	125	≥ 125	Legumes and tofu
11 Nuts	50	≥ 25	Nuts and seed, including spreads and pastes
*Added fats*			
12 Unsaturated fats	40	≥ 20	Vegetables oils/fats, except of coconut and palm oil
13 Saturated fats	11.8	≤ 11.8	Coconut oil, palm oil, and animal oils/fats (except of butter)
*Added sugars*			
14 All sugars	31	≤ 31	Sugar amount in cakes, pies, tarts, sweat rolls, patisserie, pastry, pure sugar, chocolate, confectionery, sorbet, water ice cream, soft drinks, fizzy drinks, energy drinks, isotonic drinks, diet products, sports nutrition, unspecified and mixed miscellaneous foods, and breakfast cereals

24 HDR, 24-h dietary recalls

^a^For food components which should be promoted in a healthy and sustainable diet (i.e., whole grains, vegetables, fruits, fish, legumes, nuts, and unsaturated fats) the lower consumption limit is reported, whereas for food components which should be limited (i.e., tubers or starchy vegetables, dairy foods, beef, lamb, pork, eggs, saturated fats, and added sugars) the upper consumption limit is reported [6]

^b^In the reference diet, amounts are indicated as dry, raw weights for whole grains, beans, lentils, and peas, whereas for soy foods (in our study tofu products) the non-dry weight is used [6]

^c^The amount of consumed milk products was converted into the corresponding amount of milk equivalents [27, 28]. A table displaying the conversion factors can be found in the supplementary materials (Table S4)

Whole grains were defined as food items in the category cereals having a carbohydrates-to-fiber ratio ≤ 10:1 [17] or including the word ‘Vollkorn’ (eng. ‘whole grain’) in their name. In menuCH, the amount of whole grains and legumes consumed are given as cooked weight, whereas the PHD recommendations are provided as dry weight. Therefore, conversion factors were used for the menuCH data to determine the dry weight of consumed cooked products (Table S3) [27]. In the PHD, the recommended amount for dairy products is given in milk equivalents (meq). Therefore, the amount of consumed dairy products in menuCH was converted into amounts in meq [27, 28] (Table S4). All vegetables oils/fats, except of coconut and palm oil, were allocated to the PHD food component of unsaturated fats [6]. Coconut and palm oil as well as all animal oils/fats (except of butter which was included in the dairy products food component) were included in the food component of saturated fats [6]. For the food component ‘added sugars’, the amount of consumed sugar coming from menuCH food categories with known high amount of added sugar was calculated, as in menuCH no distinction is made between added sugars and total sugars present in food. In total, 876 (6.3%) food items from menuCH food categories with known high amount of added sugar had missing information on their sugar content. Based on the Swiss [29] and French food composition database [30] missing sugar content data were added.

### Assessment of sustainability impacts

The environmental and socio-economic impacts of the Swiss diet were examined by determining GHG emissions, total land use and grassland use, the Social Hotspots Index (SHI), and the costs of the diet. Details can be found in the previous study by Frehner et al. [31].

Briefly, to assess the environmental impacts of the diet (GHG emissions, total land and grassland use), the biophysical mass-flow model SOLm [32, 33] and inventories from the Ecoinvent 3 database were used. SOLm captures all nutrient and mass flows in agricultural production of 192 countries of which only the data of Switzerland and of countries from which the produced agricultural commodities were imported to Switzerland were included in our study. Several inputs and outputs per livestock and crop activity were defined, covering all processes included in the inventories. Social risks were calculated based on the Social Hotspots Database (SHDB) [34]. The SHDB includes 156 country- and sector-specific social indicators of five main areas: labor rights and decent work, human rights, health and safety, local community, and governance. Direct impacts on the food production were determined by aggregating social indicators of 18 sub-sectors to the SHI. The indicators were weighted based on a previously established weighting scheme [34, 35]. Indirect impacts were calculated based on an input-output table for Switzerland [36] providing information on the linkages between different industries and between an industry and the final economical demand. The higher the SHI, the higher the social risk in production and throughout the value chain. Costs of a diet were calculated based on price data of 94 commodities and on food demand data [31].

## Statistical analyses

The menuCH participants’ data of the final study sample were weighted for age, sex, nationality, civil status, major living region, and household size to ensure representativeness, because they did not all have the same probability to be included in the final survey sample [19]. Season and weekday of the 24HDR interview day were additional weighting factors for variables on dietary information (see supplemental material for more details) [19].

In the main analyses, the original dietary intake cut-offs of the PHD were used, assuming a diet of 2500 kcal/day, whereas in a sensitivity analyses the cut-offs were rescaled for females to reflect a diet of 2000 kcal/day.

We used the multiple source method (MSM) [37] to calculate the habitual food intake of the study participants, using the two 24HDR and the information on food avoidance from the menuCH questionnaire. The MSM constructs a consumption distribution based on the whole study population and estimates the usual dietary intake of study participants stratified by food components [37].

The usual dietary intake amounts were used to investigate adherence of the Swiss diet to the PHD overall and by sex, language region, and dietary patterns. First, the weighted average usual intake amount per food component across the entire study population was calculated. Assuming that the target recommended daily food amount given by the PHD indicates 100%, we determined the corresponding percentage of the weighted usual intake of the average Swiss diet. Second, we estimated the proportion of study participants adhering to the single PHD food component recommendations, using the lower and upper consumption limits of the PHD instead of the target values. The target values were not used in this analysis, because they represent a population’s average diet and do not allow for substitution and compensatory effects in individual diets (e.g., allowing to interchange different animal-based and plant-based protein sources, meaning we do not expect an individual to eat red meat, poultry, fish, eggs, and lentils every day, but rather to consume only some of them or none on a given day). For food components that should be promoted in a healthy and sustainable diet (i.e., whole grains, vegetables, fruits, fish, legumes, nuts, and unsaturated fats), the lower consumption limit was used, whereas for food components which should be limited (i.e., tubers and starchy vegetables, dairy foods, beef, lamb, pork, eggs, saturated fats, and added sugars), the upper consumption limit was used. The environmental impacts were averaged for the overall population and by the subgroups sex, language regions, and dietary patterns, reporting median values.

## Results

In the overall study sample, there were more females (50.2%) than males (49.8%) (Table 2). The largest age group were the 30–44 years old (29.9%), followed by the 45–59 years old (29.8%), the 60–75 years old (21.6%), and the 18–29 years old (18.8%). Most of the participants lived in the CH-German language region (69.2%), followed by the CH-French (25.2%) and CH-Italian (5.6%) language region. The dietary pattern most frequently followed was the Swiss traditional (34.4%), while the least frequently followed dietary pattern was the Western-soft drinks (19.6%). The overall median daily energy intake was 2130 kcal with males having a higher median daily energy intake than females (2419 kcal vs. 1874 kcal).

**Table 2:** Characteristics of the menuCH study population stratified by sex (2014–2015; *n* = 2057)

Variables^a^	Overall	Males	Females
n (%)	2057 (100)	933 (49.8)	1124 (50.2)
*Age group, n (%)*			
18–29 years old	400 (18.8)	163 (17.5)	237 (20.1)
30–44 years old	533 (29.9)	237 (29.1)	296 (30.6)
45–59 years old	625 (29.8)	288 (30.6)	337 (29.1)
60–75 years old	499 (21.6)	245 (22.9)	254 (20.2)
*Language region*^b^, *n (%)*			
CH-German	1341 (69.2)	631 (71.1)	710 (67.4)
CH-French	502 (25.2)	209 (23.5)	293 (26.9)
CH-Italian	214 (5.6)	93 (5.4)	121 (5.7)
*Dietary pattern*, *n (%)*			
Prudent	486 (24.2)	165 (18.6)	321 (29.7)
Swiss traditional	744 (34.4)	289 (29.2)	455 (39.5)
Western-soft drinks	383 (19.6)	216 (24.8)	167 (14.5)
Western-alcohol	444 (21.9)	263 (27.4)	181 (16.4)
Energy intake per day [kcal]	2130 (1711, 2600)	2419 (1973, 2961)	1874 (1537, 2253)

^a^Categorical variables are expressed as n (weighted percentage (%)). Continuous variables are expressed as weighted median and weighted interquartile range. Results are weighted, as stated in the menuCH weighting strategy [19], for sex, age, major living region in Switzerland, marital status, household size, and nationality. Daily energy intake [kcal] was further weighted for weekday and season of the recall day

^b^German language region: cantons *Aargau*, *Basel City*, *Basel Country*, *Berne*, *Lucerne*, *Zurich*, and *St. Gallen*. French language region: cantons *Jura*, *Neuchâtel*, *Vaud*, and *Geneva*. Italian language region: canton *Ticino*

**Fig. 1:**
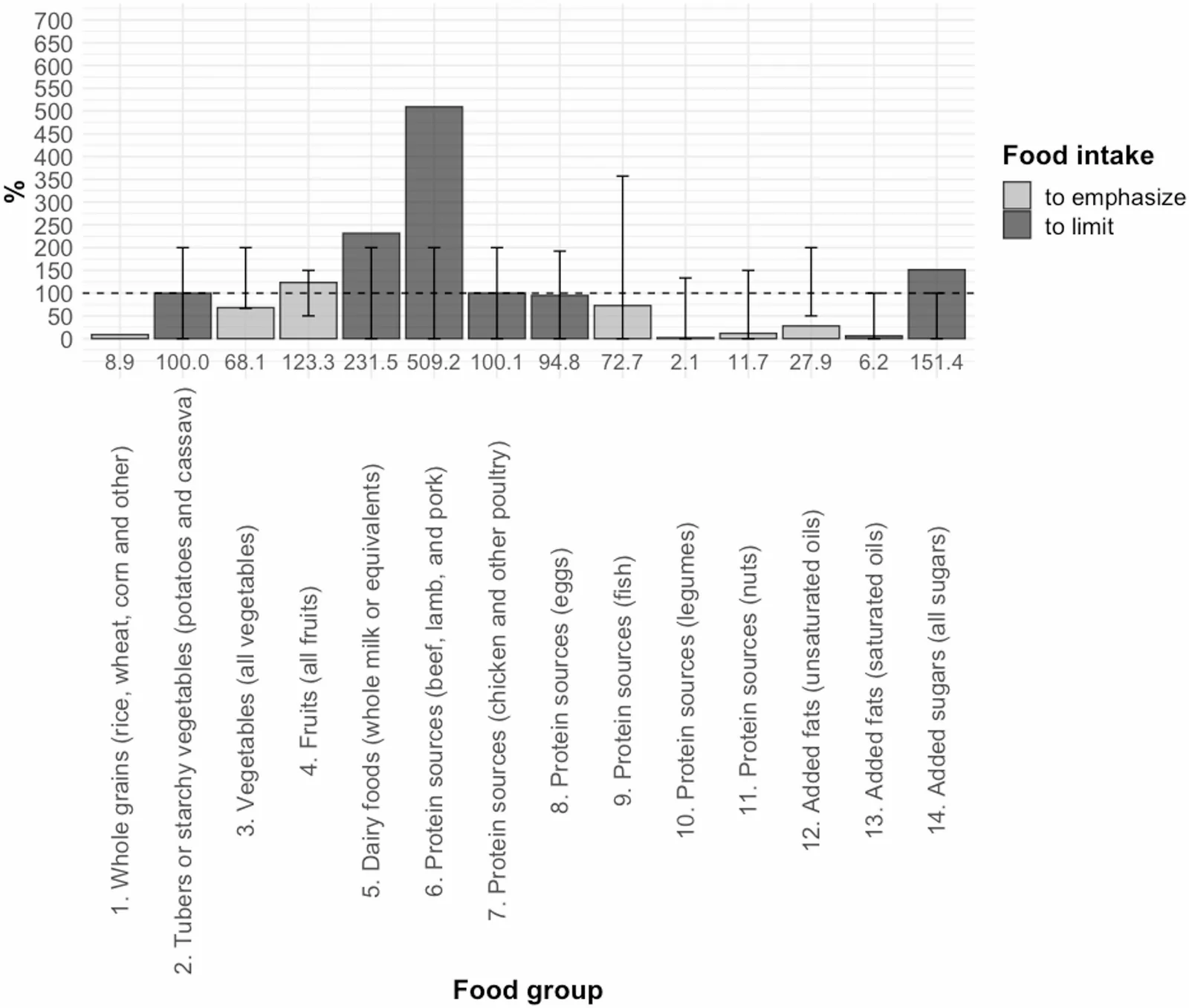
Weighted usual intake of 14 food components in the Swiss diet relative to the recommended planetary health diet consumption amount (*n* = 2057). The dotted line at 100% indicates the recommended amount by the EAT Lancet Commission, whereas the grey bars represent the weighted average usual intake of the Swiss diet with respect to the reference amount. The error bars show the percentual consumption limits of the planetary health diet in percentage

In Fig. 1 adherence to the 14 PHD food components of the whole study population is shown. The average amount of consumed starchy vegetables, fruits, dairy foods, poultry, eggs, and fish were close to the target consumption value, ranging from 72.7 to 123.3%. Low consumption was observed for whole grains (8.9%), vegetables (68.1%), legumes (2.1%), nuts (11.7%), and unsaturated fats (27.9%). In contrast, high amounts of red meat (509.2%), dairy products (231.5%), and added sugars (151.4%) were consumed in the average Swiss diet.

In Table 3, the weighted percentage of menuCH participants adhering to each of the 14 food components recommended by the PHD are shown. Overall none of the menuCH participants consumed at least 464 g of whole grains or at least 125 g of legumes per day. Next to whole grains and legumes, the least frequently met food components in the overall population were nuts (5.0%), unsaturated fats (10.9%), and red meat (14.0%), whereas the most frequently met food components were starchy vegetables (97.8%) and saturated fats (99.9%).

**Table 3:** Weighted percentage of menuCH participants adhering to 14 food components recommended by the Planetary Health Diet (*n* = 2057)^d^

	Cut-off (g)^b^	Overall (%)	Sex (%)		Language region (%)^a^			Dietary patterns (%)			
			Males	Females	German	French	Italian	Prudent	Swiss traditional	Western-soft drinks	Western-alcohol
1. Whole grains	464	0.0	0.0	0.0	0.0	0.0	0.0	0.0	0.0	0.0	0.0
2. Tubers or starchy vegetables	100	97.8	97.0	98.6	98.0	98.0	95.1	97.7	98.6	97.2	97.3
3. Vegetables	200	49.0	44.6	53.5	48.4	50.8	49.0	71.7	45.1	35.9	42.2
4. Fruits	100	82.9	78.6	87.1	83.6	82.7	74.5	89.2	87.4	77.5	73.6
5. Dairy foods^c^	500	43.2	41.6	44.8	38.7	54.5	47.0	57.2	22.3	43.0	61.3
6. Beef, lamb, and pork	28	14.0	9.6	18.3	15.3	10.8	12.0	20.1	18.4	3.9	9.4
7. Chicken and other poultry	58	94.7	92.5	96.9	94.5	95.2	95.0	93.9	95.1	94.0	95.7
8. Eggs	25	89.1	88.4	89.9	88.2	90.1	95.8	88.7	88.4	87.8	92.0
9. Fish	28	27.8	26.9	28.7	23.9	35.9	38.3	47.2	16.9	25.4	26.1
10. Legumes	125	0.0	0.0	0.0	0.0	0.0	0.0	0.0	0.0	0.0	0.0
11. Nuts	25	5.0	4.8	5.3	5.2	5.3	0.9	5.6	6.6	3.8	3.0
12. Unsaturated fats	20	10.9	12.4	9.4	10.0	10.6	23.4	20.7	6.3	7.5	10.7
13. Saturated fats	11.8	99.9	99.9	99.8	100.0	99.7	100.0	99.7	100.0	100.0	99.9
14. Added sugars	31	37.5	30.4	44.5	35.1	39.3	58.5	50.9	28.0	19.4	54.0

^a^ German language region: cantons *Aargau*, *Basel City*, *Basel Country*, *Berne*, *Lucerne*, *Zurich*, and *St. Gallen*. French language region: cantons *Jura*, *Neuchâtel*, *Vaud*, and *Geneva*. Italian language region: canton *Ticino*

^b^ For food components which should be promoted in a healthy and sustainable diet (i.e., whole grains, vegetables, fruits, fish, legumes, nuts, and unsaturated fats) the lower consumption limit was used, whereas for food components which should be limited (i.e., tubers or starchy vegetables, dairy foods, beef, lamb, pork, eggs, saturated fats, and added sugars) the upper consumption limit was used. For both sexes a diet of 2500 kcal per day was assumed

^c^ The amount of consumed milk products was converted into the corresponding amount of milk equivalents [27, 28]. A table displaying the conversion factors can be found in the supplementary materials (Table S4)

^d^ The percentages are weighted as stated in the menuCH weighting strategy [19], for sex, age, major living region in Switzerland, marital status, household size, nationality, weekday of the recall day, and season of the recall day

**Fig. 2:**
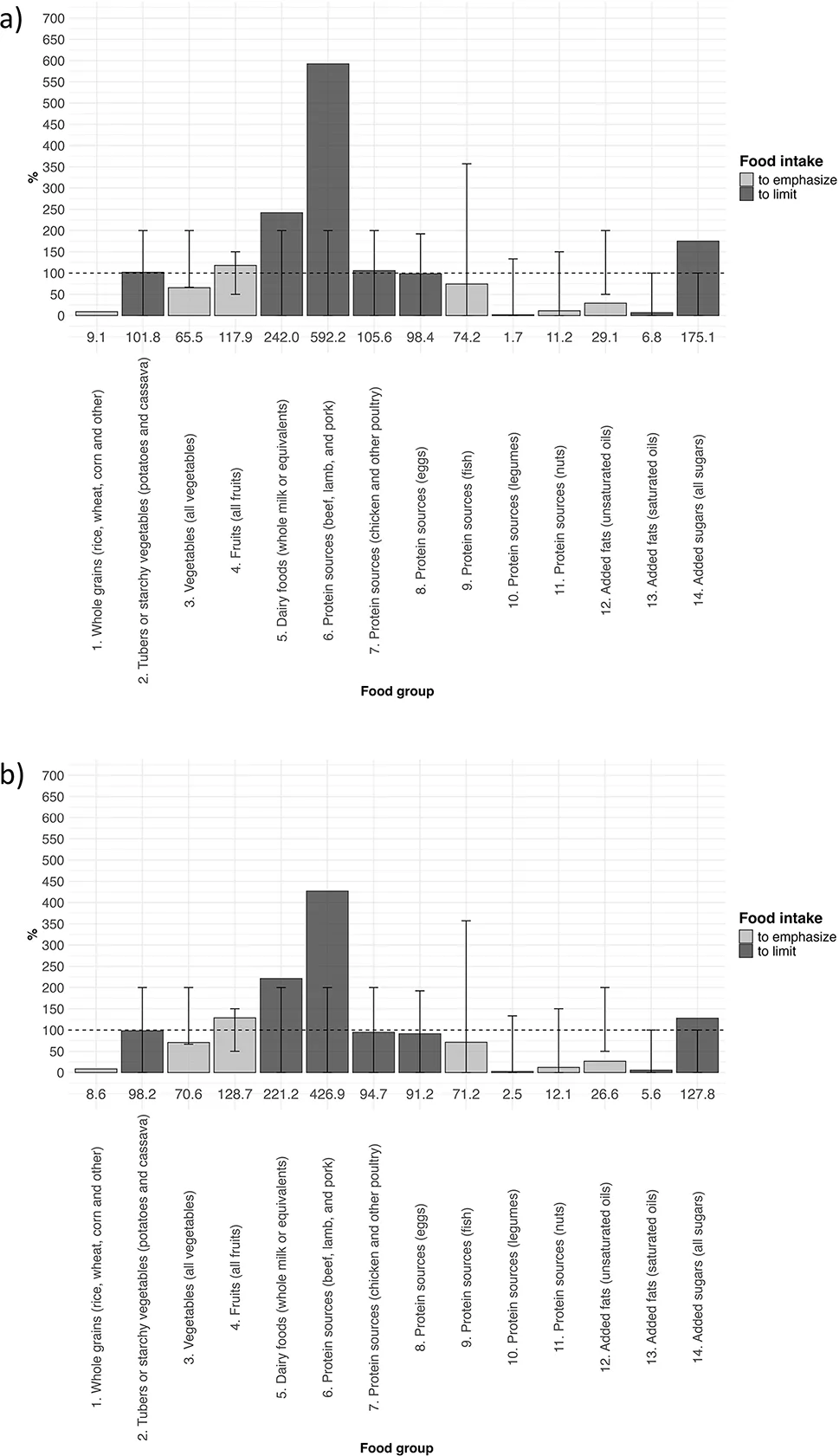
Weighted usual intake of 14 food components in the Swiss diet relative to the recommended planetary health diet consumption stratified by sex (*n* = 2057). **a** Usual intake in males, **b** Usual intake in females. The dotted line at 100% indicates the recommended amount by the EAT Lancet Commission, whereas the grey bars represent the weighted average usual intake of the Swiss diet with respect to the reference amount stratified by sex. The error bars show the percentual consumption limits of the planetary health diet in percentage

Differences in adherence to the PHD were seen among the two sexes. While females consumed on average more fruits than males, males consumed more red meat, poultry, dairy products, and added sugars than females. Twice as many females as males met the PHD recommendation on red meat consumption. Furthermore, clearly more females than males adhered to the recommendation on added sugar, i.e., limited sugar consumption. Overall, females followed the PHD more closely than males (Fig. 2; Table 3).

**Fig. 3:**
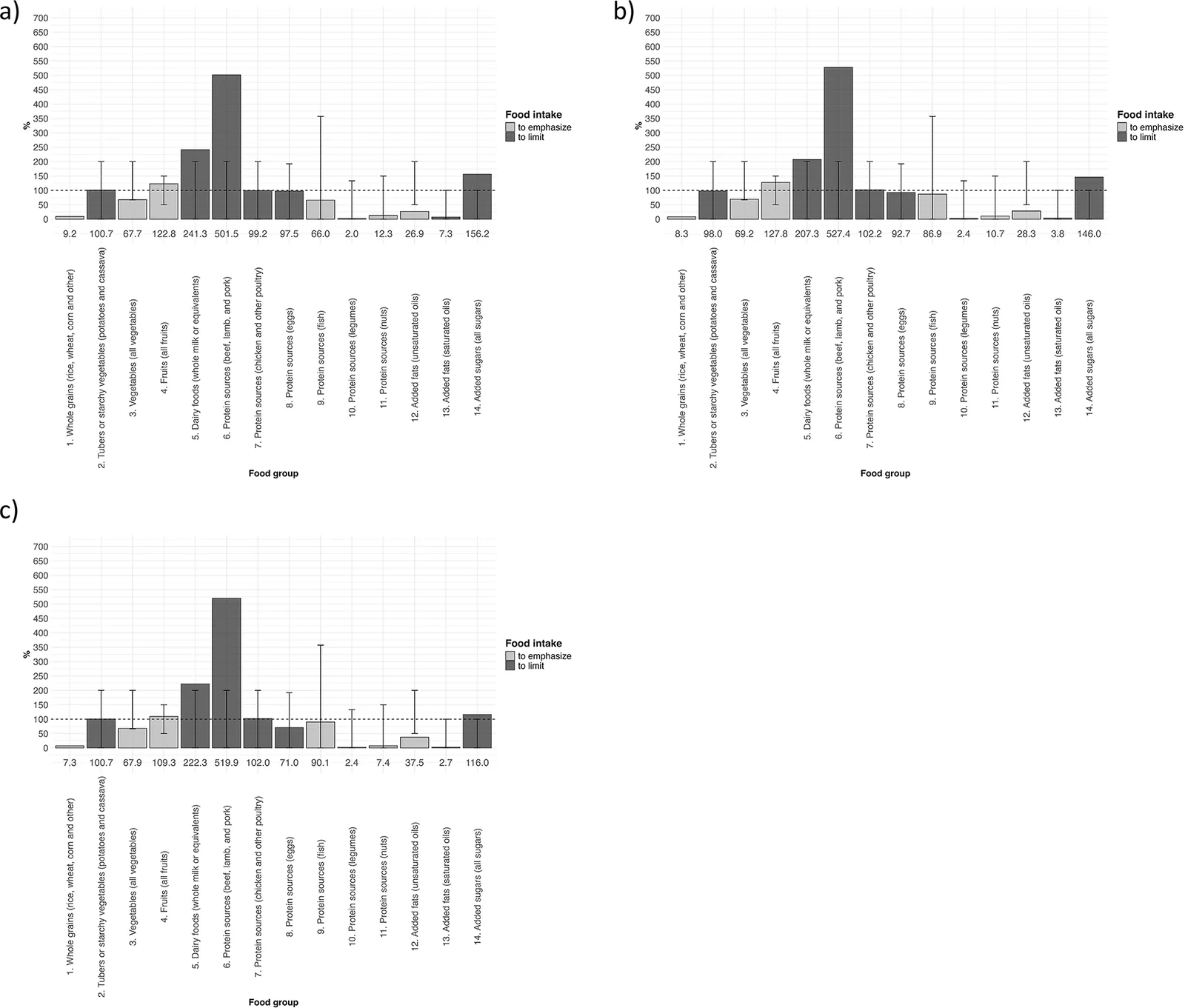
Weighted usual intake of 14 food components in the Swiss diet relative to the recommended planetary health diet consumption amount stratified by language region (*n* = 2057). **a** Usual intake in CH-German, **b** Usual intake in CH-French, **c** Usual intake in CH-Italian. The dotted line at 100% indicates the recommended amount by the EAT Lancet Commission, whereas the grey bars represent the weighted average usual intake of the Swiss diet with respect to the reference amount stratified by language region. The error bars show the percentual consumption limits of the planetary health diet in percentage

Stratifying the menuCH study population by language region revealed a higher consumption of dairy products and lower consumption of fish in the CH-German language region compared to the CH-French and CH-Italian regions. In accordance, the percentage of CH-German participants adhering to the fish and dairy recommendations was lowest. The CH-Italian language region had higher consumption of unsaturated fats and lower consumption of fruits, eggs, nuts, and added sugar compared to the CH-German and CH-French regions. Consumption of red meat was lowest in the CH-German language region, followed by the CH-Italian, and CH-French region. However, differences in adherence to the PHD across the three language regions were minimal (Fig. 3; Table 3).

Differences in adherence to the PHD were also investigated by the four main Swiss dietary patterns. In comparison to all the other dietary patterns, the prudent pattern revealed higher consumption of vegetables, fruits, fish, and unsaturated fats and a higher percentage of participants adhering to these recommendations. The Swiss traditional pattern had higher intakes of dairy products and added sugars and revealed the lowest percentage of people adhering to the dairy recommendations.

**Fig. 4:**
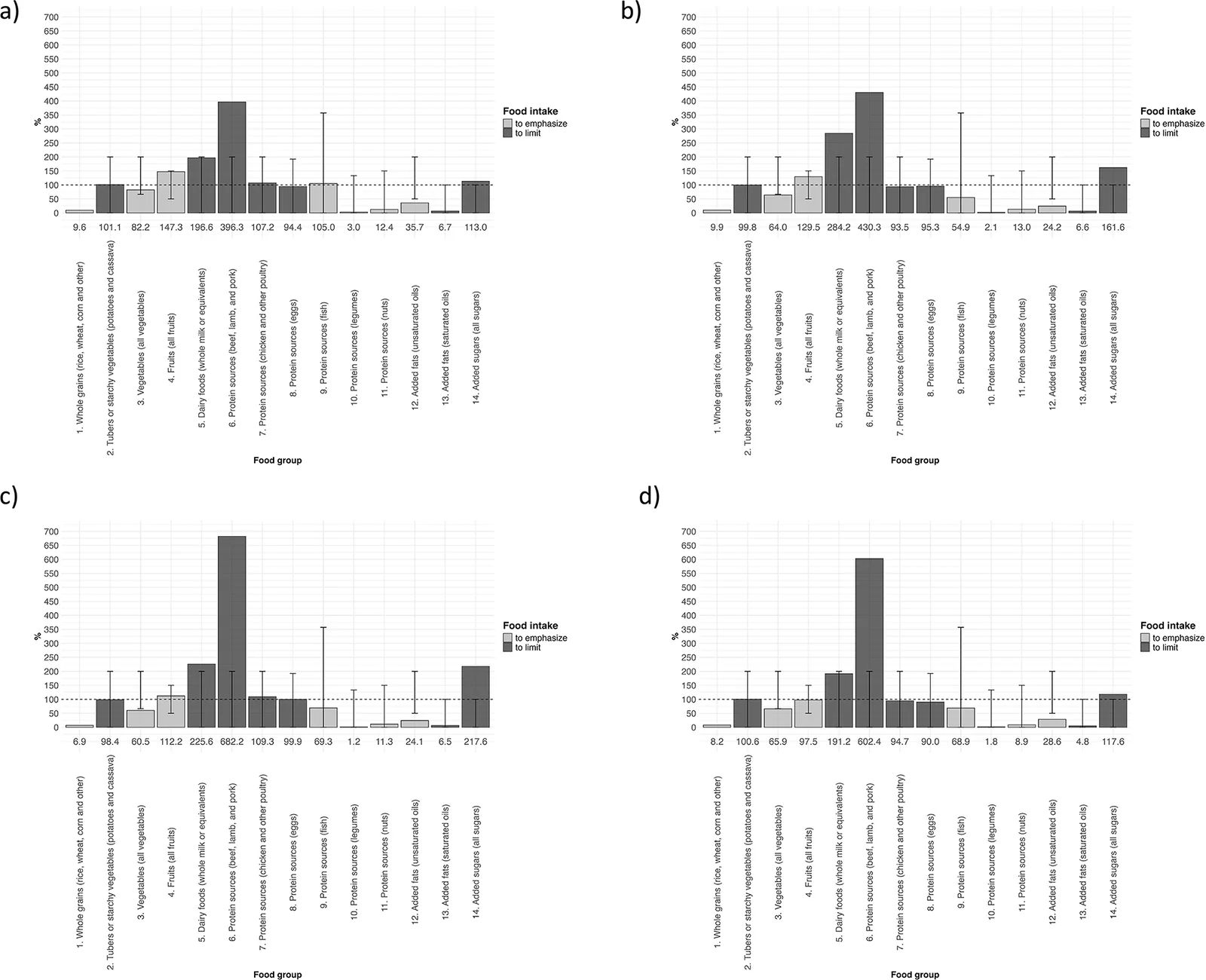
Weighted usual intake of 14 food components in the Swiss diet relative to the recommended planetary health diet consumption amount stratified by dietary patterns (*n* = 2057). **a** Usual intake in the Prudent pattern, **b** Usual intake in the Swiss traditional pattern, **c** Usual intake in the Western-soft drinks pattern, **d** Usual intake in the Western-alcohol pattern. The dotted line at 100% indicates the recommended amount by the EAT Lancet Commission, whereas the grey bars represent the weighted average usual intake of the Swiss diet with respect to the reference amount stratified by dietary pattern. The error bars show the percentual consumption limits of the planetary health diet in percentage

The Western-soft drinks dietary pattern showed higher consumption of poultry, added sugars, and red meat and the lowest percentage of participants adhering to the recommendations for the latter two food components. The Western-alcohol dietary pattern showed a high red meat consumption and had the highest percentage of participants adhering to the added sugar recommendation. Overall, the prudent dietary pattern followed the PHD the closest (Fig. 4; Table 3).

In the sensitivity analysis when adjusting the cut-off values for females to a diet of 2000 kcal per day, the results were in general similar as in the main analysis and mainly only changed when looking at the female subgroup itself. As expected, when lowering the cut-offs for females, less females adhered to the food components that should be limited, and more females adhered to the food components that should be emphasized (Table S2).

Regarding sustainability impacts, the differences observed between the subgroups were generally small, but relevant trade-offs could be identified between social impacts, costs, GHG emissions, and healthy diets (Table 4). This was most visible in the Prudent dietary pattern group, where higher risks of adverse social impacts and higher costs were seen, resulting from a higher consumption of fruits and vegetables. The higher SHI value observed in the Prudent pattern was also driven by a higher consumption of food components that are typically imported, as these foods often originate from countries with higher risks. In addition, lower consumption of animal source foods in the Prudent pattern led to lower GHG emissions and, due to a reduction in ruminants needed to produce the required animal source foods, also in lower grassland use. On the contrary, the higher fruits, vegetables, and legumes consumption resulted in a slightly higher cropland use in the Prudent pattern.

**Table 4:** Sustainability impacts of the average Swiss diet, overall and by subgroups (*n* = 2057)^a^

	Overall	Sex		Language region			Dietary patterns			
		Males	Females	German	French	Italian	Prudent	Swiss traditional	Western-soft drinks	Western-alcohol
N	2057	933	1124	1341	502	214	486	744	383	444
GHG emissions [kg CO_2_e]	3.3	3.7	2.9	3.3	3.1	3.1	3.1	3.4	3.2	3.3
Cost [CHF]	9.2	11.1	8.1	8.9	9.9	9.9	10.6	7.8	8.8	12.0
Land use [m^2^]	4.9	5.7	4.4	5.0	4.8	4.6	4.9	4.8	4.7	5.4
Grassland use [m^2^]	1.4	1.6	1.3	1.5	1.2	1.3	1.1	1.8	1.5	1.2
SHI [points]	~ 171*10^6^	~ 167*10^6^	~ 173*10^6^	~ 160*10^6^	~ 187*10^6^	~ 190*10^6^	~ 262*10^6^	~ 157*10^6^	~ 135*10^6^	~ 150*10^6^

GHG, greenhouse gas; CO2e, carbon dioxide equivalent; SHI, social hotspots index; CHF, Swiss Francs

^a^ The reported values are given as median values per person and 24 h dietary recall period

Differences also arose between males and females (Table 4). The lower values in GHG emissions, costs, cropland and grassland use were driven by a lower overall food consumption of females compared to males, and by a lower consumption of red meat and alcohol (the latter being the main driver of the higher cost value, in analogy to what was observed in the Western-alcohol dietary pattern). In contrast, the SHI was higher in females compared to males, driven by a higher consumption of plant-based products that are imported from countries with higher social risks.

## Discussion

The menuCH study population, which is representative for Switzerland, consumed on average more animal-based foods and added sugar and less plant-based proteins, whole grains, and unsaturated fats than recommended by the PHD. Stronger adherence to the PHD was seen among females and among participants following the Prudent dietary pattern. None or only a few of the participants consumed the recommended amount of whole grains and animal-based proteins, while nearly all followed the recommendations on tubers and starchy vegetables and saturated fats. The sustainability impacts were generally similar among subgroups, but relevant trade-offs were identified between social impacts, costs, GHG emissions, and healthy diets. Females had in general lower sustainability impact values than males probably due to their overall lower food intake, and lower intake of red meat and alcohol.

When comparing the average Swiss diet with the PHD, we found an underconsumption of whole grains, legumes, vegetables, nuts, and unsaturated fats and an overconsumption of red meat, dairy products, and added sugars. This was also observed in the average German [27, 38], Australian [9], Mexican [10], and Swedish [8] diets. The average Japanese diet was characterized by an overconsumption of red meat and fish and an underconsumption of nuts and dairy products [39]. Several misalignments with the PHD recommendations were also observed among residents of Mongolia [40], Brazil [41], Kenya [42], India [43], and The Gambia [7, 40], most frequently for the food components fruits, vegetables, tubers, legumes, and added sugars.

When investigating the proportion of participants adhering to the 14 food components recommended by the PHD, adherence to the whole grains, legumes, nuts, red meat, and added sugars recommendations was low, while adherence to the tubers and starchy vegetables, and saturated fats recommendations was high. Similarities were observed with two of Switzerland’s neighboring countries, France [44] and Germany [38, 44], as well as with Denmark [45] and the UK [46].

In Mexico [47] and Gambia [7], both generally considered to follow a non-Western dietary pattern, adherence to the PHD was generally lower than in Switzerland, except for a closer adherence to the whole grain recommendation in Mexico [47]. Nevertheless, a precise comparison between countries is generally difficult due to differences in the operationalization of the PHD food components in the different studies.

In our study, females adhered more closely to the PHD recommendations than males. This was also reported in several other studies [8, 41, 44, 45, 47, 48]. In the Swedish study, females showed higher score adherence for the components potatoes, fruits, red meat, and added sugar [8]. This is in line with our study, as we also observed higher adherence to the fruits, red meat, and added sugars recommendations among females than males. The Mexican study also reported higher score adherence to the tubers and red meat recommendations in females. However, adherence to the fruits recommendation was higher in males than in females [10].

Switzerland consists of multiple language regions which are culturally influenced by their large neighboring countries. Previous studies observed differences in diet and its quality by language region [14, 17], which were also observed in our study: CH-German participants consumed on average more dairy products and added sugars, but less fish and unsaturated fats than participants residing in the CH-French and CH-Italian language regions, which showed more similarities to a typical Mediterranean diet. However, general adherence to the PHD was similar among the language regions.

Among the four main Swiss dietary patterns the prudent dietary pattern, characterized by a high intake of fruits, vegetables, and fish, adhered the closest to PHD. The Swiss traditional dietary patterns, with a high consumption of dairy products, was the most frequently followed by menuCH participants. Compared to the average Swiss diet, participants following the Swiss traditional dietary pattern tended to consume more dairy products and less fish, which may reflect a lower adherence to the PHD for these components. Conversely, their lower meat consumption may indicate a somewhat higher adherence to the PHD for this component.

In line with our findings, a systematic review found evidence across 23 studies that dietary patterns with more plant-based and less animal-based foods improve health outcomes and have a lower environmental impact [49]. Similarly, a systematic review based on 76 articles concluded a lower risk of chronic disease incidence, including cancer, cardiovascular diseases, and type 2 diabetes, and of all-cause mortality with closer adherence to plant-based dietary patterns [50]. Therefore, in Switzerland, better adherence to a plant-based diet, such as the PHD, may contribute to improved population health and reduced incidence and mortality of NCDs.

The PHD was developed as a global concept. Comparisons with the PHD recommendations resulted in the average diet of many countries being too high in animal-based proteins. Nonetheless, some countries worldwide still present high rates of multiple micronutrient deficiencies and stunting, but many essential nutrients have worse bioavailability in plant source foods compared to animal source foods and reaching nutrient adequacy from plant source foods alone might be challenging [6, 51]. The strict avoidance of unprocessed red meat consumption [52] and a shortfall in essential micronutrients [51] when adhering to the PHD were criticized. In addition, previous studies have observed methodological lacks in the report, which lead to an overestimation of the decrease in NCDs mortality [53] and reduce the reproducibility and reliability of the report [54]. Meanwhile, the Global Burden of Disease study supports many of the PHD recommendations, emphasizing, however, that the dietary changes needed to decrease NCDs depend on the economic status of a country and are not universal [55]. The recommendations based on the PHD should carefully consider the country-specific context and be adapted based on the local economic and geographic situation [6].

In 2024, Switzerland has updated its food-based dietary guidelines, which now consider sustainability aspects [56]. In agreement with the PHD recommendations, the new version of the guidelines encourages the consumption of seasonal and regional fruits and vegetables, whole grain products, and plant source proteins with an emphasis on legumes consumption and deemphasis on meat intake. In addition, the consumption of nuts and plant oils is highlighted and the recommended daily amounts of milk and milk products slightly reduced. Nevertheless, the Swiss food-based dietary guidelines recommend lower amounts of whole grains, plant oils, and nuts, but higher amounts of starchy vegetables and dairy products than the PHD [56].

Regarding the sustainability impacts of the Swiss diets, low consumption of animal source products in diets was related to reduced GHG emissions and land use, while high consumption of fruits and vegetables was related to higher costs and higher risks of adverse social impacts. The latter relates to current trade patterns. Hedging against these risks would thus be possible with targeted sourcing of these products focusing on safe, fair, and just working conditions and wages in source countries of products that are typically imported in Switzerland. Sustainability impacts of food come with considerable external costs [57], and internalization, i.e. including the external costs in the price to pay, would contribute to better balance between animal- and plant-based food. In comparison to other studies, the overall per-capita daily impact values for GHG emissions (3.3 kg Carbon dioxide equivalent (CO2e)) and total land use (6.4 m2) reported in our study were at the lower end of estimates from other countries, ranging from 5.1 kg CO2e and 6.6 m2 in Italy to 5.9 kg CO2e and 7.3 m2 in France [58]. A previous Swiss study assessing the overall individual daily per capita GHG emissions for Switzerland estimated values between 3.0 and 7.1 kg CO2e [59]. The differences in these estimates are due to the methods used and the different regions investigated.

As in all observational studies assessing dietary intakes, recall bias could have led to over- or underestimation of consumed food items. Additionally, a more health-conscious study population compared to the general Swiss population cannot be ruled out. Not all 14 food components of the PHD could be operationalized exactly as described by the EAT Lancet commission, e.g., our data did not distinguish between added and total sugar amount. The menuCH survey was conducted in 2014/2015 and therefore, data used in this study reflect patterns of consumption that are approximately ten years old. However, the menuCH survey is the only national nutrition survey conducted in Switzerland and until today no other Swiss dietary data with an equal data quality and generalizability exist. In addition, dietary patterns were shown to be rather stable over time [60], and therefore, major changes in the average Swiss diet over the past ten years are unlikely. Our study had also strengths. Based on two 24HDR, we assessed the habitual dietary intake using the MSM. By applying a weighting strategy, the results of our study are representative of the Swiss population. All 14 PHD food components were operationalized and we investigated not only the overall average Swiss diet but also the diets of several subgroups of interest.

## **Conclusion**

Compared to the recommendations of the PHD, the average Swiss diet consisted of too many animal-based protein sources, such as red meat, eggs, and dairy products, and not enough plant-based foods, such as legumes and nuts. Lower consumption of animal source products would not only be beneficial for human health, but also more environmentally sustainable, as these diets tended to have reduced GHG emissions and land use. However, diets high in fruits and vegetables also showed higher costs and risks of adverse social impacts. Policy measures should focus on incentivizing the consumption of plant-based foods, especially regionally produced foods, and disincentivizing the consumption of animal-based foods in order to increase diet quality and decrease the environmental footprint of the average Swiss diet. This goal could be reached with education campaigns on diet and nutrition, starting at a young age and targeting in particular males and individuals following a Swiss traditional or a Western pattern, because they have the highest potential for improvement of their diet and therefore their health. In addition, taxation of animal-based foods and subsidies for regionally produced plant-based foods as well as sourcing plant-based foods typically imported in Switzerland from countries with safe, fair, and just working conditions and wages could be effective public health strategies to direct Swiss consumers toward a healthier and more sustainable diet.

## Supplementary Information

Below is the link to the electronic supplementary material.

Supplementary Material 1

Supplementary Material 2

## Data Availability

The data and further documents of the menuCH study, such as questionnaires and weighting strategy, are available under https://menuch.iumsp.ch.

## Abbreviations

CH: Confoederatio helvetica (lat. for Switzerland)
CO2e: Carbon dioxide equivalent
FSO: Federal Statistical Office
FSVO: Federal Food Safety and Veterinary Office
GHG: Greenhouse gas
IARC: International agency for research on cancer
ISRCTN: International standard randomized controlled trial number
MSM: Multiple source method
NA: Missing values
PHD: Planetary health diet
SHDB: Social hotspots database
SHI: Social hotspots index
WHO: World Health Organization
24HDR: 24-h dietary recalls
