# Supplementary material for: Adherence to the eat-lancet planetary health diet and sustainability impacts of the Swiss diet
Source: Eur J Nutr. 2026 May 15;65(4):134. doi: 10.1007/s00394-026-04001-y (PMC13661674; doi:10.1007/s00394-026-04001-y)
Supplement: Supplementary file 1 [file 394_2026_4001_MOESM1_ESM.docx]

**STROBE-nut: An extension of the STROBE statement for nutritional epidemiology**

Lachat C et al. (2016) STrengthening the Reporting of OBservational studies in Epidemiology – Nutritional Epidemiology (STROBE-nut): an extension of the STROBE statement. Plos Medicine 13(6) <http://dx.doi.org/10.1371/journal.pmed.1002036> [pdf](http://journals.plos.org/plosmedicine/article/asset?id=10.1371%2Fjournal.pmed.1002036.PDF) or [online](http://journals.plos.org/plosmedicine/article?id=10.1371/journal.pmed.1002036) version.

**Suter et al.; Adherence to the EAT-Lancet Planetary Health Diet and sustainability impacts of the Swiss diet**

| **Item** | **Item nr** | **STROBE recommendations** | **Extension for Nutritional Epidemiology studies (STROBE-nut)** | **Reported on page #** |
| --- | --- | --- | --- | --- |
| **Title and**  **abstract** | 1 | (a) Indicate the study’s design with a commonly used term in the title or the abstract.  The abstract indicates that this is a cross-sectional study.  (b) Provide in the abstract an informative and balanced summary of what was done and what was found.  Yes. | **nut-1** State the dietary/nutritional assessment method(s) used in the title, abstract, or keywords.  The dietary assessment method used is mentioned in the abstract. | 3-4 |
| **Introduction** |  |  |  |  |
| Background/rationale | 2 | Explain the scientific background and rationale for the investigation being reported.  We explain the scientific background and reference to previous studies in the first part of the introduction. |  | 5 |
| Objectives | 3 | State specific objectives, including any pre-specified hypotheses.  We state the aim of the study in the last paragraph of the introduction. |  | 6 |
| **Methods** |  |  |  |  |
| Study design | 4 | Present key elements of study design early in the paper.  We give details on the study design of the menuCH survey in the first subsection of the methods section (“Study design and participants of menuCH”). |  | 6-7 |
| Settings | 5 | Describe the setting, locations, and relevant dates, including periods of recruitment, exposure, follow-up, and data collection.  Information about recruitment and data collection is given in the first two subsection of the methods section (“Study design and participants of menuCH” and “Data collection in the menuCH survey”). | **nut-5** Describe any characteristics of the study settings that might affect the dietary intake or nutritional status of the participants, if applicable.  Considerations about dietary assessment are presented in the subsection “Data collection in the menuCH survey”. | 6-8 |
| Participants | 6 | a) Cohort study—Give the eligibility criteria, and the sources and methods of selection of participants. Describe methods of follow-up.  NA  Case-control study—Give the eligibility criteria, and the sources and methods of case ascertainment and control selection. Give the rationale for the choice of cases and controls.  NA  Cross-sectional study—Give the eligibility criteria, and the sources and methods of selection of participants.  Information is available in the methods subsection “Study design and participants of menuCH”. In addition, we refer to a previous publication (Chatelan et al. 2017).  (b) Cohort study—For matched studies, give matching criteria and number of exposed and unexposed.  NA  Case-control study—For matched studies, give matching criteria and the number of controls per case.  NA | **nut-6** Report particular dietary, physiological or nutritional characteristics that were considered when selecting the target population.  The population was selected from a random stratified sample intended to be representative of the adult Swiss population. No particular dietary characteristics were therefore taken into account. | 6-7 |
| Variables | 7 | Clearly define all outcomes, exposures, predictors, potential confounders, and effect modifiers. Give diagnostic criteria, if applicable.  The definition of variables considered in the analyses is given in the methods subsections “Data collection in the menuCH survey”, “The four main Swiss dietary patterns”, “Planetary Health Diet by the EAT-Lancet commission”, “Operationalization of the PHD in menuCH”, “Assessment of sustainability impacts”, and “Statistical analysis”. | **nut-7.1** Clearly define foods, food groups, nutrients, or other food components.  The food categories included in the 14 food components of the Planetary Health Diet are presented in Table 1. Further details on the operationalization of the food components of the Planetary Health Diet are given in the methods subsection “Operationalization of the PHD in menuCH”.  **nut-7.2** When using dietary patterns or indices, describe the methods to obtain them and their nutritional properties.  We refer to our previously published paper for a detailed description of the methods used to derive the four main Swiss dietary patterns (Krieger et al. 2018). | 7-12/30 |
| Data sources - measurements | 8 | For each variable of interest, give sources of data and details of methods of assessment (measurement). Describe comparability of assessment methods if there is more than one group.  The methods of assessment of the variables of interest are reported in the methods subsections “Data collection in the menuCH survey” and “Assessment of sustainability impacts”. For sustainability impacts we also further refer to a previous publication (Frehner et al. 2021). | **nut-8.1** Describe the dietary assessment method(s), e.g., portion size estimation, number of days and items recorded, how it was developed and administered, and how quality was assured. Report if and how supplement intake was assessed.  This information is presented in the methods subsection “Data collection in the menuCH survey”.  **nut-8.2** Describe and justify food composition data used. Explain the procedure to match food composition with consumption data. Describe the use of conversion factors, if applicable.  This information is presented in the methods subsections “Data collection in the menuCH survey” and “Operationalization of the PHD in menuCH”. Conversion factors used for the operationalization of the Planetary Health Diet are reported in the methods subsection “Operationalization of the PHD in menuCH”  **nut-8.3** Describe the nutrient requirements, recommendations, or dietary guidelines and the evaluation approach used to compare intake with the dietary reference values, if applicable.  The EAT-Lancet Planetary Health Diet is described in the methods subsection “Planetary Health Diet by the EAT-Lancet Commission” and “Operationalization of the PHD in menuCH”. Target and limit values of the food components of the Planetary Health Diet are presented in Table 1 and Table S1.  **nut-8.4** When using nutritional biomarkers, additionally use the STROBE Extension for Molecular Epidemiology (STROBE-ME). Report the type of biomarkers used and their usefulness as dietary exposure markers.  NA  **nut-8.5** Describe the assessment of non-dietary data (e.g., nutritional status and influencing factors) and timing of the assessment of these variables in relation to dietary assessment.  NA  **nut-8.6** Report on the validity of the dietary or nutritional assessment methods and any internal or external validation used in the study, if applicable.  Details are given in the methods subsection “Data collection in the menuCH survey” and in the Strengths and Limitations part of the discussion. | 7-10/19-20 |
| Bias | 9 | Describe any efforts to address potential sources of bias.  Information is given in the methods subsection “Statistical analyses”. Further details are presented in the Strengths und Limitations part of the discussion. | **nut-9** Report how bias in dietary or nutritional assessment was addressed, e.g., misreporting, changes in habits as a result of being measured, or data imputation from other sources  Dietary assessment data were cleaned and screened for inconsistency in the primary study (methods subsection “Data collection in the menuCH survey”). In addition, relevant missing nutrient data were completed using the Swiss and French food composition databases (“Operationalization of the PHD in menuCH”). | 10-11/19-20 |
| Study Size | 10 | Explain how the study size was arrived at.  The study size depends on the national nutrition survey menuCH. Details are given in the methods subsection “Study design and participants of menuCH”. |  | 6-7 |
| Quantitative variables | 11 | Explain how quantitative variables were handled in the analyses. If applicable, describe which groupings were chosen and why.  Details are given in the methods subsections “Operationalization of the PHD in menuCH”, “Assessment of sustainability impacts”, “Statistical analyses”. | **nut-11** Explain categorization of dietary/nutritional data (e.g., use of N-tiles and handling of non-consumers) and the choice of reference category, if applicable.  Information is given in the methods subsection “Statistical analyses”. | 9-12 |
| Statistical  Methods | 12 | (a) Describe all statistical methods, including those used to control for confounding  Information is presented in the methods subsection “Statistical analyses”.  (b) Describe any methods used to examine subgroups and interactions.  Information is given in the methods subsection “Statistical analyses”.  (c) Explain how missing data were addressed.  Information about missing data is given in the methods subsection “Operationalization of the PHD in menuCH”.  (d) Cohort study—If applicable, explain how loss to follow-up was addressed.  NA  Case-control study—If applicable, explain how matching of cases and controls was addressed.  NA  Cross-sectional study—If applicable, describe analytical methods taking account of sampling strategy.  We provide information about the weighting strategy applied in the analyses in the methods subsection “Statistical analyses” and in the supplementary material.  (e) Describe any sensitivity analyses.  We provide information about sensitivity analyses in the methods subsection “Statistical analyses”, in the results section and in Table S2 of the supplementary materials. | **nut-12.1** Describe any statistical method used to combine dietary or nutritional data, if applicable.  The use of the multiple source method to calculate the habitual food intake of the study population is described in the methods subsection “Statistical analyses”.  **nut-12.2** Describe and justify the method for energy adjustments, intake modeling, and use of weighting factors, if applicable.  Information is given in the methods subsection “Statistical analyses” and in the supplementary materials.  **nut-12.3** Report any adjustments for measurement error, i.e,. from a validity or calibration study.  NA | 10-12/14 |
| **Results** |  |  |  |  |
| Participants | 13 | (a) Report the numbers of individuals at each stage of the study—e.g., numbers potentially eligible, examined for eligibility, confirmed eligible, included in the study, completing follow-up, and analyzed.  Information is given in the methods subsection “Study design and participants of menuCH”, in the results section, in Table 2, and Figure S1.  (b) Give reasons for non-participation at each stage.  Information is given in the methods subsection “Study design and participants of menuCH”. In addition, we refer to our Figure S1 representing a participant flowchart of the menuCH survey.  (c) Consider use of a flow diagram.  A participant flowchart is provided in the Supplementary Material Figure S1. | **nut-13** Report the number of individuals excluded based on missing, incomplete or implausible dietary/nutritional data.  We only included participants with two complete 24-hours dietary recalls (see methods subsection “Study design and participants of menuCH”). | 6-7/12/32 |
| Descriptive data | 14 | (a) Give characteristics of study participants (e.g., demographic, clinical, social) and information on exposures and potential confounders  Details are given in the results section and in Table 2.  (b) Indicate the number of participants with missing data for each variable of interest  NA  (c) Cohort study—Summarize follow-up time (e.g., average and total amount)  NA | **nut-14** Give the distribution of participant characteristics across the exposure variables if applicable. Specify if food consumption of total population or consumers only were used to obtain results.  The use of food consumption data is described in detail in the methods subsection “Statistical analyses” and briefly mentioned when presenting study results. | 11-13/32 |
| Outcome data | 15 | Cohort study—Report numbers of outcome events or summary measures over time.  NA  Case-control study—Report numbers in each exposure category, or summary measures of exposure.  NA  Cross-sectional study—Report numbers of outcome events or summary measures.  Numeric results are given in the results section, presented in the figures and in Table 3 and Table 4. |  | 12-15/33-35 |
| Main results | 16 | (a) Give unadjusted estimates and, if applicable, confounder-adjusted estimates and their precision (e.g., 95% confidence interval).  Make clear which confounders were adjusted for and why they were included.  NA  (b) Report category boundaries when continuous variables were categorized.  NA  (c) If relevant, consider translating estimates of relative risk into absolute risk for a meaningful time period.  NA | **nut-16** Specify if nutrient intakes are reported with or without inclusion of dietary supplement intake, if applicable.  Information about intake of dietary supplements is given in the methods subsection “Data collection in the menuCH survey”. | 7 |
| Other analyses | 17 | Report other analyses done—e.g., analyses of subgroups and interactions and sensitivity analyses.  We report sensitivity analyses (see methods subsection “Statistical analyses”, results section and Table S2 of the supplementary materials.). | **nut-17** Report any sensitivity analysis (e.g., exclusion of misreporters or outliers) and data imputation, if applicable.  We report sensitivity analyses (see methods subsection “Statistical analyses”, results section and Table S2 of the supplementary materials.). | 11/14 |
| **Discussion** |  |  |  |  |
| Key results | 18 | Summarize key results with reference to study objectives.  Information is given in the first paragraph of the discussion section. |  | 15-16 |
| Limitation | 19 | Discuss limitations of the study, taking into account sources of potential bias or imprecision. Discuss both direction and magnitude of any potential bias.  Information is given in the last paragraph of the discussion section. | **nut-19** Describe the main limitations of the data sources and assessment methods used and implications for the interpretation of the findings.  Information is given in the last paragraph of the discussion section. | 19-20 |
| Interpretation | 20 | Give a cautious overall interpretation of results considering objectives, limitations, multiplicity of analyses, results from similar studies, and other relevant evidence.  Interpretation of results and comparison to other studies is presented in the central part of the discussion. | **nut-20** Report the nutritional relevance of the findings, given the complexity of diet or nutrition as an exposure.  Information is given in the central part of the discussion and in the concluding paragraph. | 16-21 |
| Generalizability | 21 | Discuss the generalizability (external validity) of the study results.  Information is given in the Strengths paragraphs of the discussion. |  | 20 |
| **Other information** |  |  |  |  |
| Funding | 22 | Give the source of funding and the role of the funders for the present study and, if applicable, for the original study on which the present article is based.  Information about the source of funding is given in the section “Additional Information”. |  | 7/22 |
| *Ethics* |  |  | **nut-22.1** Describe the procedure for consent and study approval from ethics committee(s).  Details are described in the methods subsection “Study design and participants of menuCH” and in the section “Additional Information”. |  |
| *Supplementary material* |  |  | **nut-22.2** Provide data collection tools and data as online material or explain how they can be accessed.  Information and references are given throughout the manuscript and in the section “Additional Information”. |  |
