# Supplementary material for: Adherence to the eat-lancet planetary health diet and sustainability impacts of the Swiss diet
Source: Eur J Nutr. 2026 May 15;65(4):134. doi: 10.1007/s00394-026-04001-y (PMC13661674; doi:10.1007/s00394-026-04001-y)
Supplement: Supplementary file 2 [file 394_2026_4001_MOESM2_ESM.docx]

**Supplementary Material**

**ADHERENCE TO THE EAT-LANCET PLANETARY HEALTH DIET AND SUSTAINABILITY IMPACTS OF THE SWISS DIET**

**Authors**

Flurina Suter^1^, Anita Frehner^2^, Adrian Muller^2^, Diego Moretti^3^, Sabine Rohrmann^1,*^, Giulia Pestoni^1,3^

**Affiliation**

^1^ Division of Chronic Disease Epidemiology, Epidemiology, Biostatistics and Prevention Institute (EBPI), University of Zurich, Zurich, Switzerland

^2^ Department of Food System Sciences, Research Institute of Organic Agriculture FiBL, Frick, Switzerland

^3^ Nutrition Group, Swiss Distance University of Applied Sciences (FFHS) / University of Applied Sciences and Arts of Southern Switzerland (SUPSI), Zurich, Switzerland

*Correspondence: Sabine Rohrmann; Division of Chronic Disease Epidemiology, Epidemiology, Biostatistics and Prevention Institute (EBPI), University of Zurich, Hirschengraben 84, 8001 Zurich, Switzerland; email: sabine.rohrmann@uzh.ch; Tel.: +41-446-345-256

Table of Contents

[menuCH weighting strategy	3](#_Toc194645843)

[Fig. S1: Participant flowchart of the menuCH survey (n = 2057, Switzerland).	5](#_Toc194645844)

[Table S1: Description of the 14 recommended food components of the Planetary Health Diet with their target value (g/day) and lower or upper range limit (g/day) based on a diet of 2000 kcal/day (for females only).	6](#_Toc194645846)

[Table S2: Weighted percentage of menuCH participants adhering to the 14 food components recommended by the Planetary Health Diet using sex-specific cut-offs (n = 2057)	8](#_Toc194645847)

[Table S3: Conversion factors to calculate dry weight of consumed cooked legumes and whole grains in the menuCH study.	10](#_Toc194645848)

[Table S4: Conversion factors to calculate consumed amount of milk products in the menuCH study in milk equivalents.	10](#_Toc194645849)

[References	11](#_Toc194645850)

# **menuCH weighting strategy**

Not all menuCH participants had the same probability to be included in the study. Therefore, the data of the final study sample had to be weighted in order to be representative for the target population. Pasquier et al. described the weighting strategy in detail. All following citations and information were taken from their study [1]. The weighting strategy consisted of three steps:

“1. Calculation of the sampling weights: The sample for the survey menuCH was selected out of the sampling frame SRPH (Stichprobenrahmen und für Personen- Haushaltserhebungen) of the Federal Statistical Office (FSO). The SRPH is the Swiss persons and households registry. The selection of subjects was carried out in five successive waves while the SRPH is updated quarterly. […] The second method is based on the assumption that the sample was selected in a single wave. [...] This method does not take into account that the survey sampling was conducted in several waves but it provides more stable sampling weights. For menuCH we finally decided to use this method.“ (pages 1-2)

“2. Correction of non-response: In view of the substantial erosion in the sample (2086 participants among the 13606 selected people), we tested whether non-response affects the population uniformly or whether certain subgroups respond better than others. The answer can be partly found when comparing the characteristics of non-participants with participants. […] The coefficients of the non-response model are shown in Table 1. It appears that nationality is the main factor associated with non-response. […] We observed that considering three classes can explain most of the variability in the non-response probability. […]” (pages 2-3)

“3. Calibration on marginal totals: The calibration consists in correcting the weights obtained after the first two steps described above to obtain identical distributions to those in the sampling frame for auxiliary variables, which are assumed to correlate with nutrition. […] The auxiliary variables used for calibration are the same as those considered in the model of non-response, i.e. age group, gender, marital status, major region, nationality and household size. […] Food consumption was assessed througth two nonconsecutive (one month apart) 24-hour dietary recalls. It is known that nutrition is correlated with seasons (spring, summer, autumn, winter) and weekdays (Mo-Th vs Fr-Su). In menuCH recalls were unevenly distributed according to these two factors. It is why we considered to calibrate the weights on seasons and weekdays (in addition to the previous auxiliary variables). We assigned the season for each participant according the mean date between his two recalls. The calibration totals for each season were determined simply by dividing by four the population total of the average sampling frame (4627878). For weekdays we considered three strata: (1) two recalls between Monday and Thursday, (2) two recalls between Friday and Sunday and (3) one recall between Monday and Thursday and one between Friday and Sunday. The calibration totals of these three strata was determined

by multiplying the population total of the average sampling frame by 16/49 , 9/49 and 24/49 respectively. Note that 28 participants had only one recall. For them, season and weekday strata were determined on the basis of a single date. In addition, one participant had no recall at all. For him, no season and weekday calibrated weight was computed. […] The weight obtained after calibration are those used for performing extrapolations to the target population for the variables of interest.” (pages 3-4)

# **Fig. S1:** Participant flowchart of the menuCH survey (n = 2057, Switzerland).

**Target Population**

(n = 4’627’878)

- Swiss resident (cantons of Aargau, Basel City, Basel Country, Berne, Jura, Neuchâtel, Lucerne, Zurich, St. Gallen, Vaud, Geneva, Ticino),
- 18 to 75 years old.

**Excluded**

- Not contacted (n = 4’614’272)

**Source Population**

(n = 13’606)

**Excluded**

- Contact was unsuccessful	(n = 7403)
- Not eligible (n = 707)

**Eligible Sample**

(n = 5496)

**Excluded**

- Non-responders (n = 3410)

**Excluded**

- Incomplete dietary assessment (n = 29)

**Study Population**

(n = 2057)

**Study Participants**

(n = 2086)

# **Table S1:** Description of the 14 recommended food components of the Planetary Health Diet with their target value (g/day) and lower or upper range limit (g/day) based on a diet of 2000 kcal/day (for females only).

| **Dietary component** | **Target amount (g/day)** | **Lower/upper amount limit (g/day)^a^** | **Food items based on menuCH 24 HDR** |
| --- | --- | --- | --- |
| **Whole grains** |  |  |  |
| 1 Rice, wheat, corn, and other^b^ | 371.2 | ≥ 371.2 | Cereals, cereal products, and grains with a carbohydrate-to-fiber ratio <= 10:1 or including ‘whole grain’ in the food item’s name |
| **Tubers or starchy vegetables** |  |  |  |
| 2 Potatoes and cassava | 40 | ≤ 80 | Potatoes and other starchy root tubers |
| **Vegetables** |  |  |  |
| 3 All vegetables | 240 | ≥ 160 | Vegetables, vegetables juices, and vegetable soups |
| **Fruits** |  |  |  |
| 4 All Fruits | 160 | ≥ 80 | Fruits and fruit juices |
| **Dairy foods** |  |  |  |
| 5 Whole milk or equivalent^c^ | 200 | ≤ 400 | Milk, milk-based desserts, cottage cheese, curd, yoghurt, sour milk products, cheese, (fermented) milk-based drinks, cream, milk-based ice cream, and butter |
| **Protein sources** |  |  |  |
| 6 Beef, lamb, and pork | 11.2 | ≤ 22.4 | Red meat including mammals, game, offal, unspecified and mixed red meat, meat products |
| 7 Chicken and other poultry | 23.2 | ≤ 46.4 | Poultry, unspecified and mixed poultry meat, and meat products based on poultry |
| 8 Eggs | 10.4 | ≤ 20 | Eggs and egg products |
| 9 Fish | 22.4 | ≥ 22.4 | Fish, seafood, amphibians, and reptiles |
| 10 Legumes^b^ | 100 | ≥ 100 | Legumes and tofu |
| 11 Nuts | 40 | ≥ 20 | Nuts and seed including spreads and pastes |
| **Added fats** |  |  |  |
| 12 Unsaturated fats | 32 | ≥ 16 | Vegetables oils/fats, except of coconut and palm oil |
| 13 Saturated fats | 9.44 | ≤ 9.44 | Coconut oil, palm oil, and animal oils/fats (except of butter) |
| **Added sugars** |  |  |  |
| 14 All sugars | 24.8 | ≤ 24.8 | Sugar amount in cakes, pies, tarts, sweat rolls, patisserie, pastry, pure sugar, chocolate, confectionery, sorbet, water ice cream, soft drinks, fizzy drinks, energy drinks, isotonic drinks, diet products, sports nutrition, unspecified and mixed miscellaneous foods, and breakfast cereals |

24 HDR = 24-h dietary recalls

^a^ For food groups which should be promoted in a healthy and sustainable diet (i.e., whole grains, vegetables, fruits, fish, legumes, nuts, and unsaturated fats) the lower consumption limit is reported, whereas food groups which should be limited (i.e., tubers or starchy vegetables, dairy foods, beef, lamb, pork, eggs, saturated fats, and added sugars) the upper consumption limit is reported [2].

^b^ In the reference diet, amounts are indicated as dry, raw weights for whole grains, beans, lentils, and peas, whereas for soy foods (in our study tofu products) the non-dry weight is used [2]. A table displaying the conversion factors can be found in the supplementary materials (Table S4).

^c^ The amount of consumed milk products was converted into the corresponding amount of milk equivalents [3,4]. A table displaying the conversion factors can be found in the supplementary materials (Table S4).

**Table S2:** Weighted percentage of menuCH participants adhering to the 14 food components recommended by the Planetary Health Diet using sex-specific cut-offs (n = 2057) ^d^.

|  | **Cut-off (g) for males^b^** | **Cut-off (g) for females^b^** | **Overall (%)** | **Sex (%)** | | **Language region (%)^a^** | | | **Dietary patterns (%)** | | | |
| --- | --- | --- | --- | --- | --- | --- | --- | --- | --- | --- | --- | --- |
|  |  |  |  | males | females | German | French | Italian | Prudent | Swiss traditional | Western-soft drinks | Western-alcohol |
| **1. Whole grains** | 464 | 371.2 | 0.0 | 0.0 | 0.0 | 0.0 | 0.0 | 0.0 | 0.0 | 0.0 | 0.0 | 0.0 |
| **2. Tubers or starchy vegetables** | 100 | 80 | 94.2 | 97.0 | 91.4 | 94.1 | 94.9 | 91.7 | 91.9 | 95.1 | 94.1 | 95.3 |
| **3. Vegetables** | 200 | 160 | 58.6 | 44.6 | 72.5 | 58.1 | 60.2 | 56.9 | 77.8 | 57.8 | 46.2 | 50.0 |
| **4. Fruits** | 100 | 80 | 84.3 | 78.6 | 89.9 | 85.0 | 84.2 | 76.4 | 90.1 | 88.5 | 80.0 | 75.1 |
| **5. Dairy foods^c^** | 500 | 400 | 35.3 | 41.6 | 29.0 | 31.6 | 44.6 | 38.1 | 46.8 | 15.2 | 35.4 | 54.4 |
| **6. Beef, lamb, and pork** | 28 | 22.4 | 11.2 | 9.6 | 12.8 | 12.0 | 9.2 | 10.5 | 16.2 | 14.6 | 2.3 | 8.3 |
| **7. Chicken and other poultry** | 58 | 46.4 | 91.6 | 92.5 | 90.8 | 92.2 | 90.5 | 89.4 | 88.2 | 92.6 | 91.3 | 94.1 |
| **8. Eggs** | 25 | 20 | 87.7 | 88.4 | 87.0 | 86.6 | 88.8 | 95.6 | 87.9 | 85.9 | 86.8 | 91.1 |
| **9. Fish** | 28 | 22.4 | 29.8 | 26.9 | 32.8 | 25.9 | 38.1 | 40.4 | 48.7 | 19.0 | 27.0 | 29.0 |
| **10. Legumes** | 125 | 100 | 0.0 | 0.0 | 0.0 | 0.0 | 0.0 | 0.0 | 0.0 | 0.0 | 0.0 | 0.0 |
| **11. Nuts** | 25 | 20 | 6.4 | 4.8 | 7.9 | 6.8 | 6.0 | 2.3 | 8.4 | 7.8 | 4.7 | 3.3 |
| **12. Unsaturated fats** | 20 | 16 | 16.3 | 12.4 | 20.2 | 15.2 | 16.4 | 29.9 | 30.1 | 10.6 | 10.1 | 16.1 |
| **13. Saturated fats** | 11.8 | 9.44 | 99.8 | 99.9 | 99.6 | 99.8 | 100.0 | 100.0 | 100.0 | 100.0 | 100.0 | 100.0 |
| **14. Added sugars** | 31 | 24.8 | 31.5 | 30.4 | 32.7 | 29.3 | 33.5 | 50.6 | 42.1 | 21.5 | 16.9 | 49.1 |

^a^ German language region: cantons *Aargau*, *Basel City*, *Basel Country*, *Berne*, *Lucerne*, *Zurich*, and *St. Gallen*. French language region: cantons *Jura*, *Neuchâtel*, *Vaud*, and *Geneva*. Italian language region: canton *Ticino*.

^b^ For food groups which should be promoted in a healthy and sustainable diet (i.e., whole grains, vegetables, fruits, fish, legumes, nuts, and unsaturated fats) the lower consumption limit was used, whereas for food groups which should be limited (i.e., tubers or starchy vegetables, dairy foods, beef, lamb, pork, eggs, saturated fats, and added sugars) the upper consumption limit was used. For males a diet of 2500 kcal per day and for females a diet of 2000 kcal per day was used as reference.

^c^ The amount of consumed milk products was converted into the corresponding amount of milk equivalents [3,4]. A table displaying the conversion factors can be found in the supplementary materials (Table S4).

^d^ The percentages are weighted as stated in the menuCH weighting strategy [1], for sex, age, major living region in Switzerland, marital status, household size, nationality, weekday of the recall day, and season of the recall day.

# **Table S3**: Conversion factors to calculate dry weight of consumed cooked legumes and whole grains in the menuCH study.

| **Food item** | **Conversion factor** |
| --- | --- |
| Legumes | 0.55 |
| Pasta and grains | 0.5 |
| Rice and Ebly | 0.33 |

# **Table S4**: Conversion factors to calculate consumed amount of milk products in the menuCH study in milk equivalents.

| **Food item** | **Conversion factor** |
| --- | --- |
| Milk | 1 |
| Milk-based desserts | 1.4 |
| Cottage cheese | 2.4 |
| Curd | 2.4 |
| Yoghurt | 1.4 |
| Sour milk products | 1.4 |
| Cheese | 7.5 |
| (fermented) milk-based drinks | 1.4 |
| Cream | 1.4 |
| Milk-based ice cream | 1.4 |
| Butter | 2.4 |

# **References**

[1]	Pasquier J, Chatelan A, Bochud M. Weighting strategy 2017. https://menuch.unisante.ch/index.php/catalog/4/download/17 (accessed November 18, 2022).

[2]	Willett W, Rockström J, Loken B, Springmann M, Lang T, Vermeulen S, et al. Food in the Anthropocene: the EAT–Lancet Commission on healthy diets from sustainable food systems. The Lancet 2019;393:447–92. https://doi.org/10.1016/S0140-6736(18)31788-4.

[3]	Schuffenhauer A, Maier M, Huber L. Auswirkungen internationaler Märkte auf die bayerische Land-und Ernährungswirtschaft Der Milchmarkt am Beispiel Käse und Butter. Schriftenreihe Der Bayerischen Landesanstalt Für Landwirtschaft 2012.

[4]	Breidenassel C, Schäfer AC, Micka M, Richter M, Linseisen J, Watzl B. The Planetary Health Diet in contrast to the food-based dietary guidelines of the German Nutrition Society (DGE). A DGE statement. Ernahrungs Umschau 2022;69:56–72. https://doi.org/10.4455/eu.2022.012.
